# The Ferroxidase–Permease System for Transport of Iron Across Membranes: From Yeast to Humans

**DOI:** 10.3390/ijms26030875

**Published:** 2025-01-21

**Authors:** Matteo Amadei, Fabio Polticelli, Giovanni Musci, Maria Carmela Bonaccorsi di Patti

**Affiliations:** 1 Department of Biochemical Sciences ‘A. Rossi Fanelli’, Sapienza University of Rome, 00185 Rome, Italy; matteo.amadei@uniroma1.it; 2 Department of Sciences, University Roma Tre, 00146 Rome, Italy; fabio.polticelli@uniroma3.it; 3 Department of Biosciences and Territory, University of Molise, 86090 Pesche, Italy; musci@unimol.it

**Keywords:** ferroxidase, multicopper oxidase, ceruloplasmin, hephaestin, ferroportin, Fet3, Ftr1, yeast, iron, copper

## Abstract

Transport of iron across the cell membrane is a tightly controlled process carried out by specific proteins in all living cells. In yeast and in mammals, a system formed by an enzyme with ferroxidase activity coupled to a membrane transporter supports iron uptake or iron efflux, respectively. Ferroxidase belongs to the family of blue multicopper oxidases, enzymes able to couple the one-electron oxidation of substrate(s) to full reduction of molecular oxygen to water. On the other hand, the permeases are widely different and are specific to Fe^3+^ and Fe^2+^ in yeast and multicellular organisms, respectively. This review will describe the yeast and human ferroxidase–permease systems, highlighting similarities and differences in structure, function and regulation of the respective protein components.

## 1. Introduction

Cells need iron for many different processes that are essential for life; however, the electron transfer properties of this metal make it potentially toxic due to its ability to react with oxygen to produce damaging reactive oxygen species (ROS). Therefore, cells have evolved a complex array of systems to tightly control intracellular iron levels. A key step is represented by directional transport of iron across the plasma membrane either to enter or to exit the cell. Various protein systems more or less specific to iron with respect to other metals that accomplish these tasks have been identified, depending on the form of iron (heme or non-heme, ferrous or ferric transporters), the concentration of the metal (high-affinity or low-affinity transporters) and the cell type.

**Figure 1:**
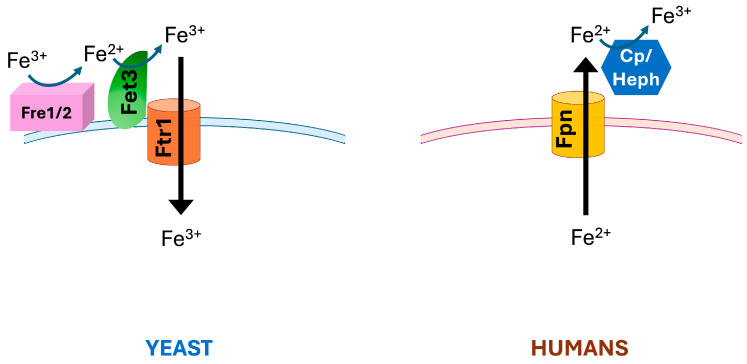
The yeast and human ferroxidase–permease system. Fpn: ferroportin; Cp: ceruloplasmin; Heph: hephaestin.

Yeast and filamentous fungi employ a system formed by an enzyme with ferroxidase activity and an iron permease for high-affinity iron uptake in conditions of metal scarcity. Intriguingly, a similar system is found in multicellular eukaryotes for export of iron from the cell (Figure 1). In both cases, the ferroxidase belongs to the family of blue multicopper oxidases (MCO), enzymes able to couple the one-electron oxidation of substrate(s) to full reduction of molecular oxygen to water. On the other hand, the iron permeases share no sequence or structural homology, and the transporter is specific for Fe^3+^ or Fe^2+^ in yeast and multicellular organisms, respectively. This bipartite system is at the basis of the well-known tight connection between copper and iron, whereby defects in copper handling translate into dysregulated iron homeostasis.

The importance of the ferroxidase–permease system is emphasised by the finding that it is among the virulence factors in pathogenic fungi and that many diseases derive from its dysregulation in humans. Mutations of the permease cause iron overload, but also insufficient ferroxidase activity leads to iron mismanagement, causing iron accumulation and oxidative stress in cells and tissues. This is particularly relevant in the brain, where iron accumulation has been linked to the onset of several neurodegenerative diseases, including the rare aceruloplasminemia and the more common Alzheimer’s and Parkinson’s diseases.

Ferroxidase–permease systems are found also in plants and invertebrates, a widespread distribution that confirms their physiological relevance. In this review we have chosen to focus on the ferroxidase–permease system of yeast and humans, highlighting similarities and differences in structure, function and regulation of the respective protein components. Molecular features specific to each protein together with aspects that are common to the yeast and human systems will be presented, to provide an overview of the knowns and unknowns of the ferroxidase–permease connection. When discussing the role of specific amino acids, residue numbering according to the full-length sequence will be used for all proteins except for ceruloplasmin (Cp). Historically, Cp amino acid numbering follows the sequence of the mature protein, without the signal peptide, and will be employed in this review.

## 2. The Ferroxidases: Structure and Mechanism

Ferroxidases allow the monoelectronic oxidation of Fe^2+^ to Fe^3+^ without the concomitant release of partially reduced oxygen species, which are potentially toxic to all organisms. These enzymes play a critical role in iron metabolism, ensuring that iron is appropriately stored, transported, and utilized. While Fe^3+^ is insoluble and therefore not easily usable as such, Fe^2+^ is soluble but prone to generate ROS through Fenton reactions [1]. Ferroxidases mitigate this toxicity by converting Fe^2+^ to Fe^3+^ before it can engage in harmful side-reactions. Consequently, these enzymes are nearly ubiquitous, with ferroxidase activities observed across bacteria, archaea, fungi, and multicellular eukaryotes, including plants.

The ferroxidases common to all living multicellular organisms are ferritins, multimeric cage-forming proteins that play a crucial role in cellular iron homeostasis, usually assembled from different kinds of chains to form a hollow, spherical architecture composed of 24 subunits [2]. While their primary function is iron storage, they achieve this goal through their ferroxidase activity, typically located in the H (heavy) chains of vertebrate ferritins or the corresponding homologous chains in other eukaryotes and bacteria. The catalytic ferroxidase center carries a high-affinity di-iron unit classically coordinated by Glu and Asp residues, with additional low-affinity iron binding sites located in close proximity [3,4,5].

In eukaryotes, the oxidation of iron from the divalent to the trivalent state is also a key step in iron transport across plasma membranes. These ferroxidases belong to the MCO family [6,7], which also includes laccases and ascorbate oxidases, and which is part of the broader class of blue copper proteins [6,8]. Their active sites typically contain four to six copper ions, classified into three types: type 1 (T1) sites facilitate electron entry from the substrate, which are subsequently transferred to a trinuclear cluster (TNC) composed of a mononuclear type 2 (T2) and a binuclear type 3 (T3) copper site. Here, oxygen binds and is reduced to water. The TNC ensures that no intermediate radical oxygen species are released during this process, thereby maintaining the safety and efficiency of the conversion. A distinctive feature of the MCO is the existence of a tight connection between the T1 site and the TNC, whereby the Cys ligand to T1 Cu is found in a highly conserved His-Cys-His sequence with the flanking His residues acting as ligands to the TNC.

The different Cu sites exhibit distinct spectroscopic features. As copper is paramagnetic in its divalent state, it can be effectively studied through electron spin resonance spectroscopy. This technique provides complex temperature-dependent line shapes that reveal valuable insight into the copper coordination geometry and the nature of the copper ligands. Additionally, in T1 sites, metal coordination with a Cys residue gives rise to charge-transfer electronic transitions that are responsible for the characteristic intense and striking blue colour of these proteins [9].

In mammals, the multicopper ferroxidases have evolved from an ancestral single-domain cupredoxin-fold protein, and comprise ceruloplasmin (Cp), hephaestin (Heph) and zyklopen (Zp) [10]. All three mammalian multicopper ferroxidases bear six copper ions, distributed into three T1 copper sites and a TNC unit, and all are involved in the facilitation of iron export by the permease ferroportin (Fpn) across plasma membranes in different tissues. A related eukaryotic ferroxidase called Fet3 is found in yeast and filamentous fungi. It shows the minimal four-copper composition, with a T1 site and a TNC. Again, it acts in concert with a permease (Ftr1) to ensure iron transport but, at variance with the mammalian ferroxidases, it is involved in iron uptake rather than export [11].

Specific structural and functional features of the yeast and mammalian blue multicopper ferroxidases will be described in the next sections.

### 2.1. The Yeast Ferroxidase Fet3

The copper–iron link, well known in higher eukaryotes, was demonstrated also in *Saccharomyces cerevisiae* when the activity of the ferroxidase-dependent, high-affinity iron uptake complex in the yeast plasma membrane was elucidated [11,12,13]. The ferroxidase Fet3 was discovered in 1994 [12]; it is a type 1 membrane glycoprotein with a large (about 550 amino acid residues) extracellular catalytic domain, a single transmembrane (TM) region and a short cytoplasmic tail. A few years later, homologs were found in fission yeast *S. pombe* [14], *C. albicans* [15] and *P. pastoris* [16], indicating conservation of the system among fungi.

**Figure 2:**
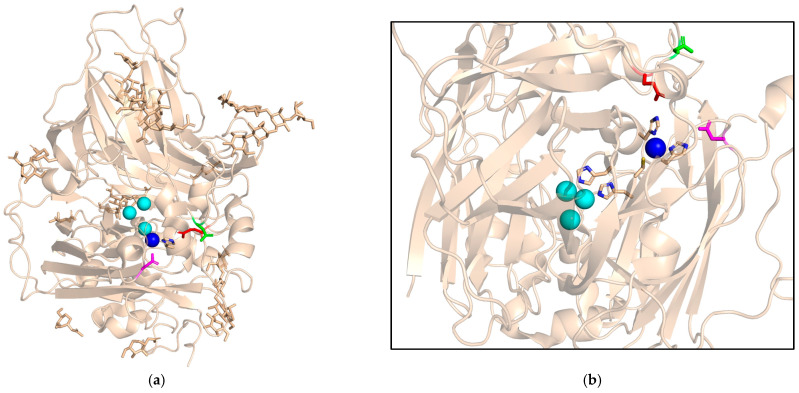
Fe binding site in Fet3 (PDB ID 1ZPU). The 3D structure of the protein is shown (**a**), together with a close-up view of the Fe-binding site (**b**). The T1 Cu atom is colored blue, the TNC is in cyan. Residues E185, D283 and D409 are colored in red, green and magenta, respectively. T1 Cu ligands and the His-Cys-His T1 Cu-TNC branched pathway are highlighted.

The three-dimensional structure of the soluble portion (i.e., lacking the C-terminal TM domain) of *S. cerevisiae* Fet3 was solved almost two decades ago [17]. In the triplicated cupredoxin fold, the single blue T1 Cu is coordinated by ligands that all belong to domain 3, whereas the TNC is found at the interface of domains 1 and 3 with eight His ligands arranged in four His-X-His sequences. The T1 copper is coordinated by H413, C484 and H489 in an approximately trigonal planar geometry [18]. As in most laccases (plant and fungal MCO essentially devoid of ferroxidase activity), the blue copper lacks the fourth ligand that pushes the coordination geometry toward a more trigonal pyramidal, as seen in Cp (see below). Instead, the particular orientation of L494 in Fet3 prevents occupancy of the apical coordination site by a donor ligand [17]. Mutagenesis of Fet3 has been extensive and has included mutations primarily restricted to the copper sites, but also to residues involved in the catalytic mechanism and in substrate specificity [19,20,21,22,23,24,25,26,27]. Before the resolution of the crystal structure, modelling studies were also of help [25,28]. This approach has highlighted the crucial role of three negatively charged residues in the catalytic activity of the protein (Figure 2).

The side chains of E185, D283 and D409 form an acidic pocket which favours the interaction with reduced iron, and are also involved in the subsequent steps of metal oxidation and transfer of electrons to the TNC [17,29]. Oxygen reduction takes place through two 2-electron steps, the first of which forms a peroxide intermediate, while the second produces the so-called native intermediate that is rapidly reduced by proton-coupled electron transfer during the catalytic cycle [30].

Kinetic analyses of the soluble truncated form of Fet3 report Km values for Fe^2+^ ranging from 4.8 μM to 7.9 μM and 4.5 μM for the enzymes from *S. cerevisiae* [31], *C. albicans* [32] and *P. pastoris* [33], respectively. Turnover values for Fe^2+^ between 50 min^−1^ at pH 6–6.5 and 1000 min^−1^ at pH 5 demonstrate wide variations in efficiency for the yeast ferroxidases [31,32,33].

The tight Mars/Venus metabolic link between iron and copper [34] can easily be seen also in the transcriptional regulation of yeast Fet3. First, the *FET3* gene is upregulated by copper. Fet3 being a copper-containing protein, this is no surprise, although it apparently also has to do with the defence against copper intoxication, since yeast lacking the *FET3* gene are highly susceptible to copper poisoning [35,36]. More interesting, the high affinity iron uptake system of *S. cerevisiae* is activated under iron deficient conditions by the transcription factor Aft1 [37], which thus acts as an iron sensor and upregulates, among others, the copper-dependent ferroxidase Fet3. Moreover, since Fet3 is not completely knocked out by Aft1 silencing, a second regulator has been invoked and identified as Ace1, also known as Cup2, a transcription factor already known as the regulator of copper detoxification genes [38].

### 2.2. The Human Ferroxidases Ceruloplasmin (Cp) and Hephaestin (Heph)

Cp was first isolated from pig and human plasma in 1948 [39], while Heph was discovered in 1999 [40] and Zp about a decade later [41]. The degree of identity among the three mammalian ferroxidases is around 50% at the amino acid level [40,41], with residues involved in copper binding and disulfide bond formation highly conserved.

Similar to Fet3, both Heph and Zp are type 1 membrane proteins with a large extracellular catalytic domain, a single TM segment and a short cytoplasmic region. The C-terminal TM domain of Heph and Zp contains 86 amino acids, with only a 23% degree of conservation at the protein level [41]. Two isoforms of Cp are found in mammals: secreted Cp is synthesized mainly by hepatocytes and released into the plasma, while a GPI-anchored form (Cp-GPI) produced by alternative splicing has been identified predominantly in the brain but also in other cell types [42,43,44]. Heph is involved primarily in intestinal iron absorption, while the role of Zp is still quite obscure [10]. Mammalian ferroxidases are large (over 1000 amino acid residues) multi-domain glycoprotein enzymes characterized by the presence of six cupredoxin domains. Cp is the only member of the family for which experimental three-dimensional structures are available, and it is the most studied of the three.

The X-ray crystal structure of Cp shows the presence of three T1 Cu binding sites located in domains 2, 4, and 6, while the TNC is found at the interface between domains 1 and 6 [45]. The T1 Cu sites in domains 4 and 6 can exist in both reduced and oxidized states; the copper atoms of these two sites are coordinated by two His residues and one Cys residue in the equatorial position and a Met residue in the axial position, giving a distorted tetrahedral arrangement. The third T1 site (in domain 2) is atypical; the Met is replaced by a non-coordinating Leu residue, similar to Fet3. However, in Cp, this T1 Cu appears stable in its reduced state and cannot be oxidized without compromising the structural integrity of the protein, suggesting that this site is likely not involved in catalysis [46].

**Figure 3:**
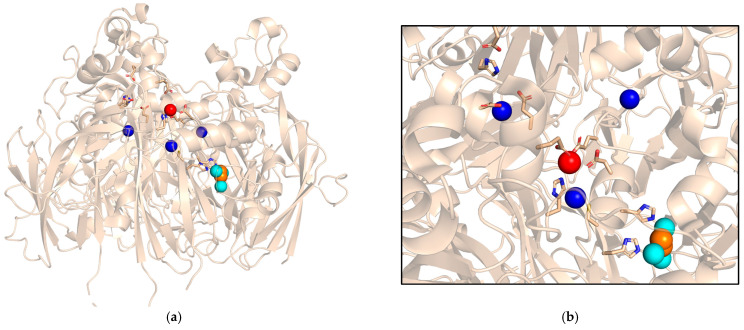
Fe binding sites in domains 4 and 6 of Cp (PDB ID 2J5W). The 3D structure of the protein is shown (**a**), together with a close-up view of the metal-binding sites (**b**). The T1 Cu atoms are colored blue, the TNC is cyan with dioxygen in orange. The metal atom in the Fe-binding site in domain 6 is in red, side chains involved in Fe binding are highlighted, together with the His-Cys-His T1 Cu-TNC branched pathway.

In addition to copper-binding sites essential for mediating electron transfer and oxygen reduction, Cp features binding sites for various substrates, including Fe^2+^, for which the enzyme exhibits the highest specificity. Based on the crystal structure solved at approximately 3 Å in the presence of Fe atoms [47], two Fe^2+^ binding sites have been identified in the protein: one in domain 6, formed by residues E935, H940, D1025 and E272 from domain 2; and another in domain 4, where iron is similarly coordinated by residues E597, H602, D684 and E971 from domain 6 (Figure 3). Additionally, two water molecules likely participate in forming these sites, which are surrounded by a negatively charged region on the surface of the protein, possibly guiding the positively charged Fe^2+^ toward them.

The Fe^2+^ binding sites are located near the T1 Cu sites in domains 4 and 6, approximately 9 Å above them. This positioning further supports the role of the T1 sites in domains 4 and 6 as primary electron acceptors, which acquire electrons directly from the substrate. In contrast, the corresponding residues in domain 2 differ (E236, Y241, N323 and E633), and Fe binding is not expected, confirming that the T1 site in this domain is not directly involved in the protein’s ferroxidase activity [46]. Site-directed mutagenesis of residues belonging to the iron-binding sites in domain 4 and 6 decreased the ferroxidase activity of Cp, confirming the functional importance of these sites [48].

Upon binding, ferrous iron transfers an electron to the closest T1 Cu site. This is coupled with the transfer of the oxidized iron to an adjacent “holding” site for ferric iron. Several pathways have been proposed for electron transfer from Fe^2+^ to the T1 Cu center in domain 6, with the most energetically efficient involving residue E272, which is directly linked via a hydrogen bond to H1026, coordinating the copper atom at the T1 site of domain 6 [49]. From T1, Cu electrons can flow via the Cys-His pathway to the TNC, where molecular oxygen binds and is reduced to water via the peroxide intermediate and the native intermediate [50], as described for Fet3.

Although the three-dimensional structure of Heph is still not available, comparative studies and molecular modelling predict a structure that closely aligns with the known structure of Cp [51,52]. Apart from the C-terminal TM region, which is not shared between the two proteins, the Heph ectodomain possesses six cupredoxin domains with six Cu binding sites, of which three are present at T1 sites distributed in domains 2, 4, and 6, while the other three form the TNC at the interface between domain 1 and domain 6, exactly as for Cp. The predicted model also shows the conservation of Cys residues involved in disulfide bridge formation and the two binding sites for Fe^2+^ that are composed of the canonical set of ligands (three acidic residues plus a His residue). Specifically, for domain 6, Heph residues E960, H965, D1050 and E300 correspond to E935, H940, D1025 and E272 of Cp. In domain 4, residues D616, H621, S703 and D996 of Heph correspond respectively to E597, H602, D684 and E971 of Cp, with the exception of the replacement D684S.

Differently from Cp, Heph T1 Cu in domain 2 has a “classical” coordination arrangement with an apical Met, and this domain is predicted to have an additional low-affinity Fe binding site composed by E264, H269, S351 and E652 [53,54]. Site-directed mutagenesis confirmed that residues E960 and H965 in domain 6 are critical for iron binding and ferroxidase activity, while the metal binding sites in domains 2 and 4 appeared to be dispensable for high affinity iron oxidation [53].

Cp exhibits two Km values for Fe^2+^: 0.6 μM and 50 μM at pH 6.5 [55], possibly corresponding to high- and low-affinity sites in domains 6 and 4 [48]. Studies on the kinetics of recombinant Heph have shown that the affinities of the two sites for iron are close to those of Cp, with two Km equal to 3.5 μM and ≈107 μM [53,56] indicating how the catalytic mechanism and the biological function of the two proteins are comparable. More recently, the kinetic constants of Cp were revised by measuring ferroxidase activity under more physiologically relevant conditions, providing Km ≈ 15 μM [57]. The catalytic efficiency of human Cp is still controversial, with published kcat for Fe^2+^ ranging from 550 min^−1^ at pH 6.5 [55] to 48 min^−1^ [50] or even less (ca. 6 min^−1^) [58] at pH 7. As regards Heph, even lower rates between 0.74 and 18 min^−1^ were reported [53,59]. However, it must be pointed out that these kcat values may be underestimates, since they were obtained on recombinant human Heph with a Cu content of 3.5–4 atoms per protein, lower than the predicted 6 Cu/protein.

Intriguingly, chloride appears to exert a modulatory action on the oxidase activity of Cp [50,60], increasing turnover at physiological pH 7 from 48 to 72 min^−1^ [50]. This effect is caused by a redistribution of electrons between T1 Cu and the TNC that accelerates intramolecular electron transfer to the TNC [50,60]. It would be interesting to verify if chloride has a similar effect on Heph. The connection between the chloride ion and ferroxidases extends to their biosynthesis, since this anion is also required for efficient loading of copper into Cp and Fet3 [61,62] (see Section 4.2).

As stated in the introductory section, malfunction of mammalian ferroxidases is associated with disease. In particular, mutations in the *Cp* gene cause aceruloplasminemia, a rare late-onset neurodegenerative pathology. Patients present iron overload primarily in the brain, but also in the liver, pancreas and retina, and they develop retinal degeneration, diabetes mellitus and neurological symptoms [63]. Missense mutations can be classified based on the type of structural and functional alterations they cause. Some mutations lead to the retention of Cp in the endoplasmic reticulum (ER) (i.e., I9F, D58H, G176R, P177R, F198S, W264S, A331D, G606E and G873E), while others result in proper secretion but produce apo-Cp, lacking copper. This occurs with mutations causing amino acid residue substitutions G631R, Q692K and G969S, which are located near the T1 Cu sites in domains 4 and 6. Another group of mutations, such as those causing Y356H and G876A substitutions, are thought to fail to stabilize FPN at the plasma membrane [64], as will be discussed in Section 4.3.

## 3. The Iron Permeases: Structure and Mechanism

Iron can cross biological membranes only via specific polytopic membrane protein carriers. While the general organization of multicopper ferroxidases is based on the cupredoxin fold and is structurally and functionally conserved across evolution, iron permeases are much more diverse in terms of structure and mechanism. In fact, proteins belonging to different families with little sequence and structural homology are responsible for the transport of ionic iron either in the ferrous or ferric form across cell membranes [65,66,67]. Ferrous iron transporters are found in evolutionarily distant organisms ranging from prokaryotes to higher-order eukaryotes; these transporters are generally quite promiscuous and while iron may be the preferred substrate, they can also transport several other divalent transition metals [66,68]. Ferrous iron transporters adopt different mechanisms and may require accessory proteins for their function. Examples are bacterial FeoB [67], the low-affinity iron transport protein Fet4 in yeast [69,70] and DMT1 (SLC11A2), together with ZIP8 (SLC39A8) and ZIP14 (SLC39A14) in mammals [65,71]. On the other hand, permeases for ionic ferric iron are much less common, show quite strict specificity for Fe^3+^ and have been found in yeast (and possibly also in some bacteria [72]) but not in mammals.

Metal-binding sites are usually formed by acidic residues Asp and Glu and may include His and less frequently other residues such as Cys or Tyr. Coordination geometry is often quite far from the ideal octahedral geometry of both Fe^2+^ and Fe^3+^, conceivably because weak binding aids efficient transport.

In general, iron transporters exploit electrochemical gradients to drive metal transport, employing different mechanisms, namely uniport, symport or antiport, which may or may not be proton-coupled. Most ferrous iron transporters are involved in iron uptake or distribution in cells and do not require a ferroxidase for their function. A notable exception is ferroportin (Fpn), the only known cellular iron exporter in mammals, which collaborates with the ferroxidases Cp or Heph to accomplish efficient efflux of the metal from cells [73]. In this setting, the ferroxidase–permease system safely generates extracellular Fe^3+^ that is then loaded onto transferrin for distribution to all tissues.

In yeast and fungi, instead, the ferroxidase–permease system captures extracellular Fe^2+^ produced by cell surface ferrireductase(s) for high-affinity uptake of Fe^3+^ in conditions of iron scarcity. The yeast permease Ftr1 shows no similarity to Fpn and no experimentally determined structure of a ferric iron permease is available to date.

Specific structural and functional features of yeast Ftr1 and mammalian Fpn will be described in the next paragraphs and discussed highlighting the molecular connection with ferroxidases.

### 3.1. The Yeast Permease Ftr1

The iron permease required for high-affinity iron uptake in yeast was first identified in *S. cerevisiae* in 1996 [11], a mere two years after the discovery of Fet3. The protein was named Ftr1 and the first evidence that Fet3 and Ftr1 cooperate and may form a complex was provided by showing that Ftr1 was required for Fet3 to acquire copper and that Fet3 was necessary for Ftr1 to reach the plasma membrane [11]. Most of our knowledge on the biology of Ftr1 has been obtained in the model yeast *S. cerevisiae*, but conservation of essential functional elements has been demonstrated in Ftr1 homologs identified and partially characterized in *S. pombe* [14], *C. albicans* [74] and *P. pastoris* [75]. The permease-oxidase system is widespread in yeast and filamentous fungi; however, despite its importance in virulence in pathogenic fungi [74,76], molecular characterization of the permease is limited to *S. cerevisiae* and *C. albicans* Ftr1.

The first studies investigating the topology of Ftr1 by addition of epitope tags in specific positions of this 404-amino acid residue protein and immunofluorescence microscopy predicted the presence of 7 TM segments with an orientation of N-terminus extracellular and C-terminus intracellular [77]. Site-directed mutagenesis identified residues crucial for the iron-transport function of Ftr1. In particular, the Glu residues in two REXLE sequence motifs in TM1 and TM4 were absolutely necessary for high-affinity iron uptake in yeast cells [11,77]. Residue E89 in TM3 and other two acidic residues in a DXXE motif in extracellular loop 6 were also demonstrated to be required for the function of Ftr1 [77]. These residues are conserved across all proteins of the Ftr1 family identified so far and their functional role has been confirmed also in *C. albicans* Ftr1 [32,78].

**Figure 4:**
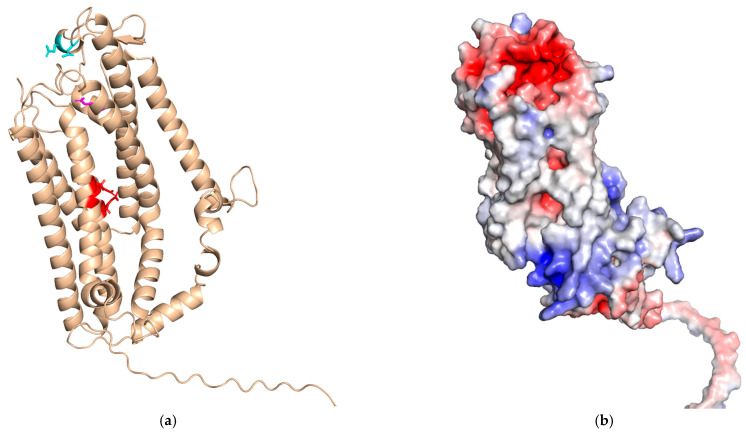
AlphaFold model of *S. cerevisiae* Ftr1 (Uniprot ID: P40088) with acidic residues of REXLE, E89 and DASE motifs evidenced in red, magenta and cyan respectively (**a**). Negatively charged surface patch on the extracellular side of Ftr1 (**b**).

Curiously, no report of the purification of Ftr1 is found in the literature, so a detailed biochemical characterization of the transport mechanism is lacking. In the absence of experimental three-dimensional structural data, the model of *S. cerevisiae* Ftr1 produced by AlphaFold [79] is shown in Figure 4.

The model is in line with available experimental data and predicts that the two REXLE motifs form a negatively charged channel within the core of Ftr1 embedded in the membrane. E89 and the DXXE acidic residues would be placed in close proximity, forming a negative patch on the extracellular side of the protein above the central channel. This arrangement is suggestive of a pathway for iron from Fet3 to Ftr1.

A first indication that Fet3 and Ftr1 may form a complex came from the finding that co-expression of *S. pombe* ferroxidase fio1 and permease fip1 in a *S. cerevisiae fet3Δ* strain restored high-affinity iron uptake, while fio1 alone was unable to complement lack of Fet3 [14]. Furthermore, co-IP demonstrated in vivo physical association between Fet5 and Fth1, the components of a second ferroxidase–permease system, responsible for iron mobilization from the vacuole in *S. cerevisiae* [80]. No cross-interaction with Fet3 and Ftr1 was found [81], indicating specificity of recognition between the two ferroxidase–permease pairs. Chemical cross-linking captured a complex between *P. pastoris* Fet3 and Ftr1, indicating that also in other yeast species the ferroxidase and permease are strongly associated [75].

A physical association between *S. cerevisiae* Fet3 and Ftr1 has been demonstrated with a series of elegant experiments [81,82]. Two-hybrid analysis allowed pinpointing of sequence elements in both proteins that were required to support an interaction. In particular, extracellular loop 6 containing the DASE (246–249) motif of Ftr1, and the C-terminal cytoplasmic domains of both proteins were identified. Within the cytoplasmic regions, residues RRGH (317–320) of Ftr1 and YGMM (581–584) of Fet3 were found to be necessary for interaction and correct trafficking to the plasma membrane [81]. Taking advantage of fully functional fluorescent fusions Fet3–CFP and Ftr1–YFP, further indication of close proximity of Fet3 and Ftr1 was obtained by in vivo Fluorescence Resonance Energy Transfer (FRET) [81].

High-affinity iron uptake shows Km values below 0.5 μM in *S. cerevisiae* [82] and *S. pombe* [14]. In *P. pastoris* a higher Km of 1.5 μM was measured, but Vmax rates were similar to those of *S. cerevisiae* despite the much higher kcat of *P. pastoris* Fet3, suggesting the ferroxidase reaction was not the rate-limiting step [16]. Uptake is strictly dependent on iron oxidation, as exogenous Fe^3+^ is not translocated by Ftr1. Kinetic analyses of high-affinity iron uptake evidenced a Fet3–Ftr1-dependent iron channeling transport mechanism in *S. cerevisiae* [82] and *C. albicans* [32]. Demonstration of iron channeling from Fet3 to Ftr1 was provided by performing iron uptake measurements in the presence of ferric iron chelators of varying binding strength. Lack of correlation between inhibition by chelators and their strength is taken as an indication that Fe^3+^ is not released to bulk solvent but directly transferred from Fet3 to Ftr1. Replacement of Fet3 E185 (E185D) and Ftr1 acidic residues in extracellular loop 6 (DASE to NASQ) had a synergic effect on the double mutant protein complex, suggesting that E185 and the DXXE motif may simultaneously bind Fe^3+^ along the pathway [82].

### 3.2. The Human Permease Ferroportin (Fpn)

**Figure 5:**
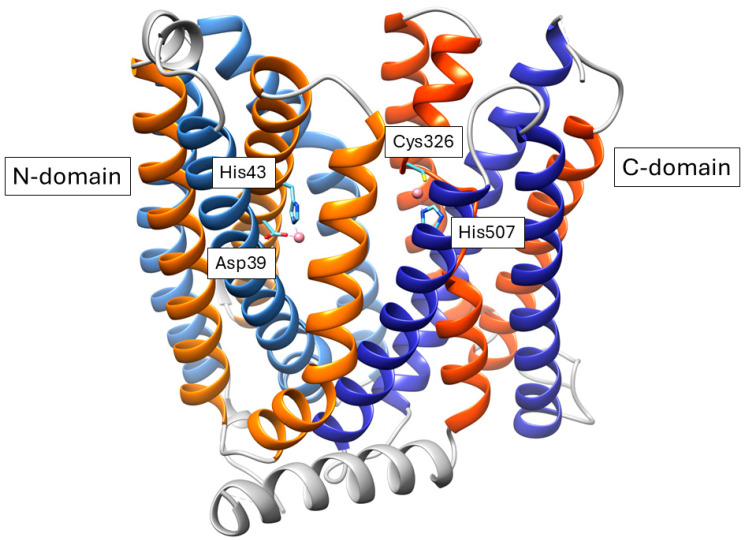
Schematic representation of the three-dimensional structure of human Fpn in complex with Co^2+^ (PDB ID 8Dl8). The first and second repeat of the N-domain are in orange and light blue, respectively. The corresponding repeats of the C-domain are in red and blue. The large intracellular region connecting the two domains is in gray. Metal binding residues are shown in stick representation, and the cobalt ions are shown as pink spheres.

Fpn, an integral membrane protein located on the plasma membrane, is the only known human iron exporter [83,84]. Its discovery was reported by three independent groups in 2000 [85,86,87], ending the hunt for the mammalian counterpart of Ftr1. Since 2010, prediction of human Fpn structure revealed that it displays the typical fold of Major Facilitator Superfamily (MFS) members [88,89,90], which was later confirmed by the cryo-EM structure [91]. This fold is characterized by 12 TM helices, organized in clearly distinct N-terminal and C-terminal domains, connected by a long loop on the cytoplasmic side. From an evolutionary viewpoint, each domain is the result of the assembly of inverted 3 + 3 helical repeats, whereby helices 1, 2 and 3 are evolutionary related to helices 4, 5 and 6, and helices 7, 8 and 9 to helices 10, 11 and 12 [92,93] (Figure 5).

The Fpn transport mechanism, as for other MFS members, has been described as an alternating access or “rocker-switch” mechanism in which the transporter cycles through inward-open, occluded and outward-open states. The required conformational changes take place through a rigid-body relative rotation of the N-terminal domain with respect to the C-terminal one, with distinct sets of interactions (mainly salt bridges) connecting the extracellular ends of the two domains in the inward-open conformation and the intracellular ends in the outward-open conformation [65,92,93].

The exact details of the iron export mechanism are still a matter of debate, with contrasting evidence supporting a Fe^2+^/2H^+^ antiport mechanism as opposed to a Fe^2+^/H^+^ symport [94,95]. Structural studies of mammalian Fpn evidenced the presence of two metal binding sites [91,94,96,97], the first composed by D39 and H43 and the second by C326 and H507 (Figure 5). However, the mechanisms driving iron-binding, conformational transition and iron release are far from being understood.

In addition, data have been reported regarding the involvement of the cytoplasmic iron chaperone PCBP2 (poly(rC) RNA binding protein 2) [98]. This is a “moonlighting” protein formed by three KH (KH1, KH2 and KH3) domains, which in its iron loaded form has been shown to physically interact with the C-terminal end of Fpn through its KH2 domain [99]. Further, it has been demonstrated that iron efflux is strictly dependent on an extracellular ferroxidase activity which acts as a “bridge” to load Fe^3+^ onto the serum iron transporter transferrin [87,100]. This activity is provided either by Heph, at the basolateral membrane of enterocytes [101] and in neurons [102], or by Cp, in astrocytes [43], macrophages [44] and other cell types [73].

In analogy with the yeast system, formation of a ferroxidase–permease complex has also been predicted for Fpn with Cp or Heph. However, demonstration of such a complex is still indirect and inconclusive, implying the physical interaction may be weaker. Blue native/SDS-PAGE suggested interaction between rat Fpn and Heph [103]. A physical association between Fpn and Heph has been shown by FRET analyses of fluorescently tagged Fpn-CFP and Heph-YFP co-expressed in HEK293 cells [104]. As regards Cp, the only indication of the existence of a complex with Fpn was provided by co-IP of Cp-GPI and Fpn in mouse astrocytes [43]. Kinetic analyses of the mammalian ferroxidase–permease system are limited, due to scarcity of tractable experimental models. Most studies have assessed the properties of wild type or mutant Fpn alone, either reconstituted in liposomes or overproduced in various cells lines. Reported Km values for transport of Fe^2+^ by human Fpn in liposome-based assays range from 0.077 μM [95] to 13.6 μM [91]. Production of recombinant Fpn in *Xenopus* oocytes loaded with radioactive ^55^Fe suggested a sub-micromolar Km [105]. In this experimental system, stimulation of metal efflux rates in the presence of externally added soluble Cp, with or without transferrin, was also observed [87,105].

Mutations causing replacement of Fpn key residues involved in iron binding and transport, and in ubiquitination and internalization of the transporter are found in patients affected by type 4 haemochromatosis, an iron overload pathology [106,107]. Loss of function mutations, i.e., those that impair the iron transport activity or the correct folding, or the membrane targeting of the protein, lead to hemochromatosis type 4A. This is characterized by iron overload, mainly in reticulo-endothelial macrophages, and low transferrin saturation. Examples of loss of function mutations are those affecting G80, D84 and R88, residues building up the so-called Motif A, essential for the conformational transition from the inward-open to the outward-open of the transporter [65,108]. Gain of function mutations, i.e., those that impair the mechanism of Fpn internalization and degradation mediated by the peptide hormone hepcidin, give rise to hemochromatosis type 4B. This is characterized by liver hepatocytes iron overload and high transferrin saturation [106,107]. Almost all the gain of function mutations cause amino acid residue replacements grouped around the hepcidin binding site, located in between TM2 and TM11 in the outward open conformation of Fpn [91,94]. These mutations involve residues Y64, A69, S71, V72, N144, C326, Y501, D504 and H507 [109].

## 4. Biosynthesis, Trafficking and Post-Translational Regulation

All yeast and human ferroxidases and permeases are integral membrane proteins, except for Cp, which is either produced as a soluble isoform that circulates in plasma or is membrane-associated by addition of a GPI anchor. The yeast and human systems share common steps during biosynthesis and trafficking through the secretory pathway to reach their final destination in the plasma membrane. Post-translational modifications include glycosylation and, specific to the ferroxidases, copper loading.

In the next paragraphs, similarities and differences between the yeast and human ferroxidase–permease systems regarding trafficking, copper loading and post-translational regulation will be described.

### 4.1. Trafficking and Glycosylation

The biosynthesis of the ferroxidases follows the classical secretory pathway: co-translational translocation, directed by the signal peptide, targeting the protein to the ER, where the initial steps of N-glycosylation occur; subsequently, transfer to the Golgi and then to the plasma membrane take place. The permeases lack a signal peptide but follow the same route to the plasma membrane.

In yeast, the tight connection between the ferroxidase and the permease is further demonstrated by the finding that assembly of the complex takes place very early in the ER and is necessary for correct processing and subcellular localization of both Fet3 and Ftr1. Ftr1 is found in the ER in the absence of Fet3 [11]. Similarly, in the absence of Ftr1, Fet3 is mis-localized to the ER in a steady state by a mechanism that requires the retrieval receptor Rer1 [110]. It has been suggested that S567 in the TM region of *S. cerevisiae* Fet3 is critical for recognition by Rer1 and this residue is masked by the interaction of Fet3 with Ftr1, releasing the complex from Rer1-dependent recycling between the ER and the Golgi and allowing plasma membrane targeting [110]. Cell biology studies indicate that Fet3 and Ftr1 are maintained at the plasma membrane through retromer-mediated retrograde transport in conditions of iron scarcity [111]. A sorting signal in the cytoplasmic tail of Ftr1 (amino acid residues 319–328) is required for endocytic recycling as opposed to entry into a degradative pathway [111].

On the other hand, there is no indication that the ferroxidase and the permease reciprocally influence their trafficking during biosynthesis, and they appear to be very much independent in humans. This may not be surprising because Fpn cooperates with different ferroxidase partners, depending on the cell type, so correct plasma membrane localization has to be guaranteed regardless of which ferroxidase is expressed by the cell. Furthermore, in some districts, such as the blood-brain barrier, Fpn may rely on soluble Cp rather than membrane-associated Cp-GPI or Heph [112]. Instead, a role for Cp and Heph in the stabilization of Fpn at the cell surface has been observed and will be described in Section 4.3.

Glycans are dispensable for enzymatic activity, but they are necessary for stability and correct trafficking of the ferroxidases. *S. cerevisiae* Fet3 has 13 potential N-glycosylation sites mostly in domains 1 and 2, 11 of which show glycan units in the X-ray structure [17]. Comprehensive deletion analysis of glycosylation sites allowed identification of N194 (positioned in a cleft between domains 1 and 2) as essential for ER exit and localization of Fet3 to the plasma membrane [113]. Cp has seven potential N-glycosylation sites in its amino acid sequence. Studies on Cp purified from plasma have confirmed that residues N119, N339, N378 and N743 are modified by the addition of bi- and tri-antennary oligosaccharide chains, while N208, N569 and N907, located in β-strands within hydrophobic regions, are not glycosylated [114,115]. These three unmodified sites are conserved in Heph while another five potential N-glycosylation sites (N164, N714, N758, N829 and N873) are not conserved. Heph is glycosylated, but the nature and location of oligosaccharide chains are currently unknown [116].

Both yeast and human permeases present a single potential N-glycosylation site in an extracellular loop, at N254, close to the DASE motif for *S. cerevisiae* Ftr1, and at N434 for human Fpn. The glycosylation site is not conserved in yeast permeases and no indication of actual addition of a glycan chain is available. On the other hand, N434 is conserved and demonstration that Fpn is glycosylated has been provided, although lack of the oligosaccharide chain was shown to have no impact on the function of mouse Fpn [117]. Whether glycans play a role in ferroxidase–permease complex formation is currently unknown.

### 4.2. Copper Incorporation

Although the ferroxidases can traffic to the plasma membrane or the extracellular space independently of copper loading, this process represents a crucial step for activation of the enzymes, which takes place in a late-Golgi compartment [118,119]. Following copper uptake, the metal is delivered by a cytosolic Cu chaperone to a P-type ATPase copper pump that transfers it in the lumen of the Trans-Golgi Network for incorporation in recipient cuproenzymes. The proteins involved in this process are strikingly conserved between yeast and humans: the human copper pump ATP7B can functionally replace yeast Ccc2 to activate Fet3 [120,121,122] and, vice versa, yeast Ccc2 is able to supply copper for loading into Cp [123]. Another feature common to both yeast and humans is the requirement of chloride for efficient copper loading of Fet3 [61] and Cp [62]. Chloride channels Gef1 in yeast and ClC-4 in hepatocytes are anion-proton exchangers that localize to a late-Golgi compartment and promote copper incorporation in the ferroxidases, possibly by counteracting acidification of the vesicle lumen [62,124]. Chloride was also shown to be an allosteric effector of copper loading for Fet3, and it was suggested that it may bind to the protein [61], in analogy to Cp.

The molecular details underlying Cu incorporation by the ferroxidases remain unknown. A convenient way to assess metal loading is Cu-dependent oxidase activity, which can be easily detected by non-denaturing SDS-PAGE both for Fet3 [13] and Cp [125]. This technique also allows distinguishing of the apo- and holo-protein on the basis of their different electrophoretic mobility. Studies in yeast demonstrated that the copper pump Ccc2 is essential to produce enzymatically active Fet3 [118,126]. Partially saturated forms of Fet3 lacking either T1 Cu (C484S) or T2 Cu (H81Q) can be produced by removal of specific ligands by site-directed mutagenesis, allowing dissection of the contribution of the different Cu sites to the stability and reactivity of the protein [22,26,27,127].

On the other hand, the Cp metalation process is considered “all or nothing”, as partially copper-saturated forms have never been identified [119,128]. The structural integrity of the five loops connecting the globular domains of Cp is crucial for proper metal loading by apo-Cp; despite their low sequence homology, these loops all start with a CX(R/K) motif, stabilized by disulfide bridges formed by the Cys residues. These loops are similarly arranged at the base of the protein. Mutations in Cys, Arg, or Lys residues render Cp inactive, preventing it from acquiring copper from ATP7B, trapping it in the Golgi, or leading to its secretion as apo-Cp. One hypothesis suggests that these inter-domain loops are necessary for proper interaction between apo-Cp and the short loops connecting the transmembrane domain with the luminal domain of ATP7B, from which Cu is released [129]. The copper incorporation process for Heph is likely to be similar to that of Cp, but it has not been studied in the same detail [104,116].

Both Cp and Heph apoproteins are substantially less stable than the holoproteins, showing increased sensitivity to proteolysis, which reflects conformational differences between the apo-and holoproteins [116,119,130]. The half-life of circulating apo-Cp is about 5 h compared to 5.5 days for holo-Cp [128]. Mature apo-Heph lacking Cu is correctly localized at the plasma membrane, but it is unstable and is rapidly degraded by the proteasome following ubiquitination [116].

Given the conservation of the proteins involved in copper delivery to the ferroxidases, yeast has proven to be an excellent model for studies of pathogenic defects in the copper loading machinery and more generally of alterations in copper homeostasis. Examples are the characterization of human ATP7B mutations causing Wilson’s disease [122,131] or the use of yeast models of rare neurodegenerative diseases to specifically investigate the copper/iron interplay [132].

### 4.3. Post-Translational Regulation

Intracellular iron levels must be tightly controlled to avoid both overload and deficiency, conditions that are detrimental to cell survival. Transcriptional and post-transcriptional regulation is effective to control biosynthesis of the ferroxidase–permease system [73,133]; in addition, protein localization at the cell surface is also directly regulated by various post-translational mechanisms. Removal of the permease rather than the ferroxidase is more efficient to shut down iron transport, making the transporter the primary target of regulatory mechanisms.

Iron levels control the abundance of the ferroxidase–permease system at the cell surface, though by different means in yeast and humans. External high iron induces internalization of a transport-active Fet3–Ftr1 complex to an early endosome and sorting to the vacuole for degradation [134,135]. The iron-dependent signal that mediates this process is unknown. Ftr1 is ubiquitinated, probably after iron-regulated endocytosis because internalization appears to take place independently of ubiquitination [135], marking the complex for degradation.

Analogously, transport-active Fpn is targeted for degradation in conditions of iron excess. Hepcidin, the liver-produced peptide hormone that is the master regulator of systemic iron homeostasis in mammals [136], binds to Fpn, inducing internalization, ubiquitination and degradation of the permease [137,138,139]. The peptide also blocks iron efflux by occluding Fpn, and it has been found that hepcidin binding is coupled to iron binding, with an 80-fold increase in hepcidin affinity in the presence of iron [91]. A model for hepcidin regulation of Fpn in which only Fpn molecules loaded with iron are targeted for degradation has been proposed [91].

The ferroxidases Cp and Heph both play a role in maintaining Fpn at the cell surface [102,140], rather than assisting in permease trafficking, as happens in yeast. It has been demonstrated that the ferroxidase activity of Cp is required for the stability of cell surface Fpn and the transporter is rapidly internalized and degraded in the absence of Cp [140]. Furthermore, Cp can partially prevent internalization of Fpn mediated by hepcidin, while a set of aceruloplasminemia mutants are ineffective [64]. It has also been reported that in the presence of ferroxidase activity, Fpn is less sensitive to hepcidin in human brain microvascular endothelial cells [100,112]. Hence, Cp (and probably also Heph) contributes two functions to support Fpn-mediated iron efflux: one is ferroxidase activity that would rapidly remove the metal from the transporter and the other is stabilization of Fpn at the plasma membrane through an as yet unknown mechanism, possibly by interfering with binding of hepcidin and/or by locking Fpn in a conformation that cannot be ubiquitinated.

Copper deprivation affects the ferroxidase–permease system by causing production of apo-ferroxidase devoid of enzymatic activity. In yeast, this inactivates the system but has a limited effect on abundance of the complex at the plasma membrane [35,81]. In mammals, instability of apo-Cp or apo-Heph together with lack of ferroxidase activity substantially reduce the amount of Fpn at the cell surface, as outlined above.

## 5. Conclusions

After discovery of the Fet3–Ftr1 system in yeast, the identification of Fpn established the partnership of the mammalian ferroxidases Cp and Heph and brought the human system to center stage. Much is now known on both systems and, as often happens, the simpler yeast Fet3–Ftr1 complex is providing clues (and new questions) that are contributing to our understanding of the copper–iron connection in eukaryotes. For example, in yeast, a tight macromolecular Fet3–Ftr1 complex where Fe^3+^ can be directly channelled from Fet3 to Ftr1 and into the cell is optimized to prevent precipitation of oxidized iron before it crosses the membrane. Whether a channeling mechanism is effective also for Cp-Fpn (and Heph-Fpn) is worth probing.

Structural biology data for both the yeast and human ferroxidase–permease complex is lacking and molecular details of how the two proteins interact are unknown. Stoichiometry may be assumed to be 1:1, but there is evidence that Fpn may be dimeric [141,142], although this has been questioned [143,144], adding to the complexity of the system.

To quote the authors of the paper that showed that fungal Fet3 can increase plasma iron in aceruloplasminemic mice, “the direction of iron movement is not specific to the multicopper oxidase but rather is determined by the acceptor of the ferroxidase reaction” [145]. The extracellular ferric iron generated by the Cp–Fpn or Heph–Fpn system is delivered to transferrin; nothing is known about the intracellular Fe^3+^ acceptor in yeast. The possibility that ferroxidase–permease systems are actually part of “iron-handling super-complexes” involving other donors and acceptors is fascinating and is beginning to be explored [146].

## Data Availability

No new data were created or analyzed in this study.
